# METTL3 mediates bone marrow mesenchymal stem cell adipogenesis to promote chemoresistance in acute myeloid leukaemia

**DOI:** 10.1002/2211-5463.13165

**Published:** 2021-05-20

**Authors:** Zhi‐peng Pan, Bin Wang, Di‐yu Hou, Ruo‐lan You, Xiao‐ting Wang, Wen‐hui Xie, Hui‐fang Huang

**Affiliations:** ^1^ Central Laboratory Fujian Medical University Union Hospital China; ^2^ Clinical Laboratory Fujian Maternal and Child Health Hospital Fujian Children's Hospital China; ^3^ Graduate School Fujian Medical University Fujian Medical University Union Hospital China

**Keywords:** adipogenesis, AML, chemoresistance, m^6^A, MSCs

## Abstract

Adipogenesis of bone marrow mesenchymal stem cells (MSCs) promotes chemoresistance of acute myeloid leukaemia (AML) cells. MSCs from AML patients (AML‐MSCs) display enhanced adipogenesis compared with bone marrow MSCs from healthy donors. However, the precise molecular mechanism by which adipogenesis of MSCs from AML marrow differs from normal counterparts remains obscure. We found that METTL3 significantly inhibits MSC adipogenesis. Here, we aimed to identify the molecular mechanism linking METTL3 and MSC adipogenesis. Analysis of m^6^A epigenetic changes in MSCs determined via RIP‐qPCR and MeRIP‐qPCR indicated that METTL3 affects AKT protein expression in MSCs by mediating m^6^A modification of *AKT1‐mRNA*. Downregulated METTL3 expression in AML‐MSCs induced an increase in AKT protein, resulting in enhanced MSC adipogenesis, thereby contributing to chemoresistance in AML cells. Therefore, targeting AKT regulation by mRNA modification in MSC adipogenesis might provide a novel therapeutic strategy to overcome AML chemoresistance.

## Graphical abstract

We find that METTL3 reduces the level of m^6^A modification of AKT‐mRNA transcripts, which prevents reader protein‐mediated AKT‐mRNA decay, thereby increasing mRNA stability and protein expression. Activation of the PI3K/AKT pathway promoted adipogenic differentiation of MSCs, thereby contributing to chemoresistance of AML cells. This study provides new insights into AML chemoresistance and may benefit the treatment of AML.

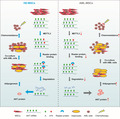

**Abbreviations**
AML: acute myeloid leukaemia
AML‐MSCs: MSCs from AML patients
Ara‐C: cytarabine
BMM: bone marrow microenvironment
BP: biological process
CCK‐8: Cell Counting Kit‐8
DNR: daunorubicin
HD‐MSCs: MSCs from healthy donors
KEGG: Kyoto Encyclopedia of Genes and Genomes
m^6^A: N6 methyladenine
MNCs: mononuclear cells
MSCs: mesenchymal stem cells
ORO: Oil Red O
qPCR: quantitative PCR

Acute myeloid leukaemia (AML) cells remain in intimate contact with the stromal microenvironment in the bone marrow (BM) [1]. Remodelling the bone marrow microenvironment (BMM) promotes the development of AML [2, 3, 4]. Mesenchymal stem cells (MSCs) are the precursors of osteoblasts and adipocytes in BM and are an important component of the BMM [2]. The balance between osteogenesis and adipogenesis ensures normal BM function [5, 6]. BM MSCs from patients with AML (AML‐MSCs) weaken osteogenesis and enhance adipogenesis [7], which promotes the development of leukaemia [8].

N^6^‐methyladenine (m^6^A) is the most abundant and reversible RNA modification in eukaryotic mRNA [9, 10]. Methyltransferase complexes (writers), usually including METTL3, METTL14 and WTAP, demethylases (erasers), such as ALKBH5 and FTO, and m^6^A reader proteins (readers), such as the YTH and IGF2BP families, modify RNA with m^6^A [11, 12, 13]. These proteins can reversibly regulate RNA splicing, transport, translation and stability [11, 12, 13]. The methylase METTL3 can participate in the regulation of stem cell self‐renewal and differentiation [14]. For instance, METTL3 is abundantly expressed in AML and induces the progression of leukaemia in mice transplanted with AML [15]. Meanwhile, the demethylase FTO can reduce the levels of ASB2 and RARA m^6^A‐mRNA and promote oncogene‐mediated malignant transformation and leukaemia [16]. In addition, m^6^A methylation regulates the process of fat formation by mediating mRNA splicing [17] and the expression of adipogenesis‐related proteins [17]. Abundant METTL3 expression inhibits the adipogenesis of MSCs derived from porcine BM [18], whereas knocking out METTL3 in healthy mouse BM MSCs enhances adipogenesis [19]. However, the role of METTL3 in the adipogenesis of human AML‐MSCs remains unclear.

Here, we investigated the role of METTL3 BMSC adipogenesis to deepen the understanding of the regulatory mechanisms of m^6^A methylation in adipogenesis with respect to modulating AML chemoresistance. Our results of RNA sequencing (RNA‐seq), m^6^A microarray analysis and validation using clinical specimens showed that METTL3, *AKT‐mRNA* and AKT‐mRNA m^6^A were differentially expressed in MSCs from healthy donors (HD‐MSCs) and AML‐MSCs. These findings offer a theoretical basis for determining new therapeutic targets for AML from the perspectives of m^6^A and the BMM, which have important clinical value.

## Materials and methods

### AML cell lines and primary BM MSCs

Human AML cell lines HL‐60, U937 and THP‐1 from Cell Bank of Type Culture Collection Chinese Academy of Sciences (Shanghai, China) were cultured and maintained in RPMI 1640 medium (HyClone, Logan, UT, USA) containing 10% FBS (Gibco, Life Technologies, Grand Island, NY, USA), 2 mm l‐glutamine (Gibco), 100 units/mL of penicillin and 100 µg·mL^−1^ of streptomycin (Gibco) under a humidified 5% CO_2_ atmosphere at 37 °C. Human AML‐MSCs derived from BM specimens were obtained from the Haematology Department of the Fujian Medical University Union Hospital. This study was conducted in accordance with the Declaration of Helsinki (2013) for experiments involving humans and was approved by the ethics committee of Fujian Medical University Union Hospital. Written informed consent was obtained from all patients before participation. Table 1 shows the clinical information of the patients. BM mononuclear cells (MNCs) were isolated using Ficoll‐Hypaque density centrifugation [20]. The MNCs were seeded into low‐glucose Dulbecco's modified Eagle medium (LG‐DMEM; HyClone) supplemented with 20% FBS, 100 units/mL of penicillin and 100 µg·mL^−1^ of streptomycin, and cultured in a humidified 5% CO_2_ atmosphere at 37 °C for 3 days. Nonadherent cells were removed, and then, adherent MSCs were passaged at 90% confluence and expanded to passage 4 (P4). BM‐derived HD‐MSCs (Cyagen Biosciences, Santa Clara, CA, USA) were identified as CD34^−^/CD44^+^/CD45^−^/CD73^+^/CD90^+^/CD105^+^ types with positive rates > 95%. The MSCs differentiated into osteocytes and adipocytes confirming their multidirectional differentiation potential.

**Table 1:** Clinical information of AML patients and healthy donors. All samples were obtained at diagnosis. FAB, French–American–British classification; WBC: white blood cell count.

Sample ID	Gender	Age	WBC (10^9^/L)	FAB
P1^a^	Female	19	40.48	M5
P2	Male	28	16.44	M5
P3	Female	25	36.87	M2a
P4	Male	30	30.75	M5b
P5	Male	37	106.40	M5b
P6	Female	41	87.68	M5
P7	Male	45	35.89	M2
P8	Male	29	13.30	M5
P9	Male	33	427.80	M1
P10	Male	29	283.96	M5
P11	Male	34	295.64	M5b
P12	Male	34	70.46	M2a
P13	Male	28	10.94	M5
P14	Male	45	19.66	M5
P15	Male	48	62.67	M5
P16	Male	42	69.98	M5b
P17	Female	31	23.87	M5b
P18	Male	30	4.06	M5
P19	Male	48	39.39	M5
P20	Female	44	58.18	M2
P21	Female	27	12.29	M2
P22	Female	22	1.61	M2
P23	Male	39	17.47	M2b
H1^b^	Male	31	4.90	Healthy donor
H2	Male	26	7.68	Healthy donor
H3	Male	33	5.23	Healthy donor
H4	Female	31	3.95	Healthy donor
H5	Female	29	7.59	Healthy donor

^a^ Patient.

^b^ Healthy donor.

### MSC adipogenesis and Oil Red O staining

We cultured MSCs in alternating adipogenic medium A (DMEM supplemented with 10% FBS, 0.5 mm 3‐isobutyl‐1‐methylxanthine, 1 mm dexamethasone, 10 mm glutamine and 5 mg·mL^−1^ insulin [Cyagen Biosciences]) for 3 days, with adipogenic medium B (DMEM supplemented with 10% FBS, 10 mm glutamine and 10 mg·mL^−1^ insulin), for 1 days until adipocytes appeared. The differentiation process continued for 14 days, when lipid droplets became obvious indicating differentiation into adipocytes, which were then stained with Oil Red O (ORO; Millipore Sigma Co., Ltd., Burlington, MA, USA) [21, 22]. Briefly, purified cells were fixed with methyl alcohol for 3 min and then incubated with Giemsa stain for 20 min at room temperature. The cells were washed with water and air‐dried, and then stained for 10 min with a filtered working solution of 0.35% ORO stain in isopropanol to ddH_2_O (3 : 2). The cells were rinsed three times with distilled water and examined using a microscope (Leica Microsystems GmbH, Wetzlar, Germany). We quantified triglyceride accumulation by eluting ORO‐stained lipids with 100% isopropanol and then measuring optical density at 450 nm by spectrometry (Thermo Fisher Scientific Inc., Waltham, MA, USA).

### MSC osteogenesis and Alizarin Red S staining

The MSCs (2 × 10^5^ /well) were cultured with 0.1% gelatine in six‐well plates. When the ratio of fusion reached between 60% and 70%, the medium was discarded and osteogenic differentiation culture medium (2 mL) was added. The cells were fixed in 4% paraformaldehyde 21 days after the induction of differentiation for 15 min [21, 23] and then stained with 1% Alizarin Red (AR; Sigma‐Aldrich Corp., St. Louis, MO, USA) in 10% cetylpyridinium chloride at pH 4.2 for 5 min. Thereafter, optical density was assessed at 562 nm using a spectrometer (Thermo Fisher Scientific Inc.) [24].

### Adipocyte differentiation induced using MK‐2206 2HCL

MK‐2206 2HCL (HY‐10358, MCE) was dissolved in DMSO at a stock concentration of 10 mm and diluted to 4 μm in adipogenic media A and media B. The MSCs were incubated for 72 h with adipogenic medium A and adipogenic medium B for 24 h. Adipogenic medium A and medium B were alternated until adipocytes appeared. The differentiation process continued for 12 days before staining with ORO.

### Co‐cultivation and chemoresistance assays

Differentiated adipocytes were washed twice with normal growth medium and then placed in the same medium. Thereafter, AML cells (U937, HL‐60 and THP‐1; 5.0 × 10^5^/well each; 6‐well plates) were co‐cultured with differentiated adipocytes in RPMI1640 for 24 h, and then, the sensitivity of AML cells to chemotherapy was measured using Cell Counting Kit‐8 (CCK‐8) as described [25]. Briefly, AML cells were seeded into 96‐well plates in growth medium and incubated with 100 ng·mL^−1^ daunorubicin (DNR; Xinshidai, Shanghai, China), 10 μm cytarabine (Ara‐C; Cytosar, Foshan, China) or PBS (vehicle control). The cell growth inhibition rate (%) was calculated as follows:

$$\frac{1‐(\mathrm{experimental}\;\mathrm{group}‐\mathrm{blank}\;\mathrm{control})}{\left(\mathrm{control}\;\mathrm{group}‐\mathrm{blank}\;\mathrm{control}\right)}\times 100\%$$

### RNA m^6^A quantitation

Total RNA was isolated using TRIzol (Invitrogen Corp., Carlsbad, CA, USA) as described by the manufacturer. The quality of RNA was analysed using a NanoDrop^™^ Spectrophotometer (Thermo Fisher Scientific Inc.). The m^6^A content in total RNA was quantified using EpiQuik m^6^A RNA Methylation Quantification Kits (Epigentek, Farmingdale, NY, USA) [26]. Briefly, wells were coated with 200 ng of RNA, and then, capture and detection antibodies at suitable concentrations were added separately to the wells as described by the manufacturer. The m^6^A levels were quantified by reading the absorbance of each well at 450 nm to create a standard curve [27]. All samples were assessed in triplicate.

### RNA extraction, complementary DNA (cDNA) synthesis and quantitative (q)PCR

Total RNA was isolated using TRIzol (Invitrogen) as described by the manufacturer. Total RNA (200 ng) was reverse‐transcribed in 10 μL reaction volumes using cDNA Synthesis Kits (Roche Holdings AG, Basel, Switzerland), as described by the manufacturer. Quantitative PCR (qPCR) was performed using the cDNA as template, SYBR Green qPCR Master Mix (Roche, Mannheim, Germany) and a model 7500 Real‐Time PCR System (Applied Biosystems, Waltham, MA, USA). The endogenous controls were GAPDH or ACTB, and each reaction was run in triplicate. The qPCR comprised 1 μL of cDNA, 5 μL of SYBR Green qPCR Mix, 0.3 μL of PCR primers and 3.4 μL of RNase‐free water. The cycling protocol was 95 °C for 1 min, followed by 35 cycles of 95 °C for 15 s, 60 °C for 30 s and 72 °C for 30 s. The respective forward and reverse primers were as follows (5′→3′):

METTL3: CAGGGCTGGGAGACTAGGAT and CTGGGCTGTCACTACGGAAG; AKT1: TGAGGAGCCCTGGTCTAATGAT and AAGACCCATTCAGAAGAGTTAT; c‐MYC: CCTACCCTCTCAACGACAGC and TTGTTCCTCCTCAGAGTCGC; and GAPDH: GGAGCGAGATCCCTCCAAAAT and GGCTGTTGTCATACTTCTCATGG. Relative expression *in vitro* and *in vivo* (clinical) was analysed using the 2^–ΔΔCt^ method [28], and three independent replicates of all biological samples were assessed.

### Western blotting

Proteins were extracted using radioimmunoprecipitation assay buffer (Thermo Fisher Scientific Inc.) containing protease and phosphatase inhibitor cocktails (Thermo Fisher Scientific Inc.), and then, protein concentrations were determined using BCA Protein Assay Kits (Thermo Fisher Scientific Inc.). Protein (30–60 μg/well) was resolved by 10% SDS/PAGE, transferred onto polyvinylidene fluoride membranes (Thermo Fisher Scientific Inc.) and activated with methanol. The membranes were washed with Tris‐buffered saline plus Tween 20, nonspecific protein binding was blocked with 5% milk, and then the blots were incubated with 1 : 1000‐diluted primary antibodies against METTL3 (Abcam, Cambridge, UK; #ab195352), GAPDH (Cell Signaling Technology, Danvers, MA, USA; #5174), AKT (Cell Signaling Technology; #4691), p‐AKT (Cell Signaling Technology; #4060) and PPAR‐γ (Abcam; #ab178860). Secondary antibodies were 1 : 5000‐diluted HRP‐conjugated goat anti‐mouse or rabbit IgG (H + L) as appropriate (Beyotime Institute of Biotechnology, Nanjing, China). Chemiluminescence signals were visualised using BeyoECL Star Kits (Beyotime Institute of Biotechnology) and detected using the ChemiDoc Touch System (Bio‐Rad Laboratories Inc., Hercules, CA, USA). Band intensity was quantified using imagej software (National Institutes of Health, Bethesda, Maryland).

### Production of lentiviral particles and infection of MSCs

The wild‐type METTL3 coding region sequence (METTL3‐CDS) was amplified from HD‐MSC cDNA using PCR and the respective forward and reverse primers (5′→3′): AAATCTAGAATGTCGGACACGTGGAGCTC and TTTGCGATCGCCTATAAATTCTTAGGTTTAGAG. The PCR‐amplified product was subsequently cloned into pLJM1‐EGFP (Brett Stringer; RRID: Addgene_19319; http://www.addgene.org/19319/), verified by DNA sequencing and transfected into 293T cells. The negative control was a nontargeting empty pLJM1‐EGFP vector. The sequences of short hairpin RNAs (shRNAs) targeting human METTL3 (shRNA #9 and shRNA #12) were used as described [15]. The control was scramble shRNA (Addgene_1864; http://www.addgene.org/1864/). Thereafter, MSCs were infected with lentiviral particles. The effects of METTL3 overexpression and knockdown were confirmed by RT‐qPCR and western blotting.

### Microarray hybridization and relative data analysis

Immunoprecipitated RNA samples of the HD‐MSCs and AML‐MSCs were labelled with Cy5 fluorescent dye using Super RNA Labelling Kits (Arraystar Inc., Rockville, MD, USA) and then purified using RNeasy Mini Kits. The Cy5‐labelled cRNAs were fragmented and hybridised to a human mRNA and lncRNA m^6^A epitranscriptomic microarray (8 × 60 K; Arraystar) containing 44 122 mRNA and 12 496 lncRNA degenerate probes. The hybridised arrays were scanned using a G2505C Scanner (Agilent Technologies Inc., Santa Clara, CA, USA) [29]. All spots on the microarray were evaluated using Feature Extraction Software Version 11.0.1.1 (Agilent Technologies Inc.). The raw intensity of immunoprecipitated RNAs was normalised using an average of log_2_‐scaled spike‐in RNA intensities. The fold changes between the HD‐MSCs/AML‐MSCs were determined for each transcript, and *P*‐values were calculated. Differentially m^6^A‐methylated RNAs were identified using a cut‐off of fivefold (*P* < 0.05). Differentially m^6^A‐methylated mRNA transcripts were identified using Gene Ontology, Kyoto Encyclopedia of Genes and Genomes (KEGG) pathway analyses and Gene Set Enrichment Analysis (GSEA) [30].

### Sequence‐based RNA adenosine methylation site predictor (SRAMP) database

The SRAMP can predict m^6^A modification site characteristics [31]. The full‐length RNA sequence of AKT1 was entered into SRAMP to predict possible positions of m^6^A modifications on AKT1.

### RNA‐binding protein immunoprecipitation (RIP) assay

Immunoprecipitated RNA‐binding protein was assayed using Magna RIP Kits as described by the manufacturer (Millipore Sigma Millipore Sigma Co., Ltd.). Harvested MSCs were lysed with RIP lysis buffer on ice and then incubated with the input anti‐METTL3 (Abcam), anti‐m^6^A (Synaptic Systems GmbH, Göttingen, Germany) and anti‐IgG at 4 °C overnight. The RNA complexes were extracted using proteinase K and phenol/chloroform/isoamyl alcohol, amplified by qRT‐PCR.

### Statistical analysis

Data are expressed as means ± SD of three independent experiments. All data were analysed using graphpad prism version 8.0 (GraphPad Software Inc., La Jolla, CA, USA). The significance of differences between groups was determined using Student *t*‐tests, and values with *P* < 0.05 were considered statistically significant.

## Results

### Sensitivity of AML cells to chemotherapy decreased after co‐cultured with differentiated adipocytes due to the enhanced adipogenesis of MSCs

**Fig. 1:**
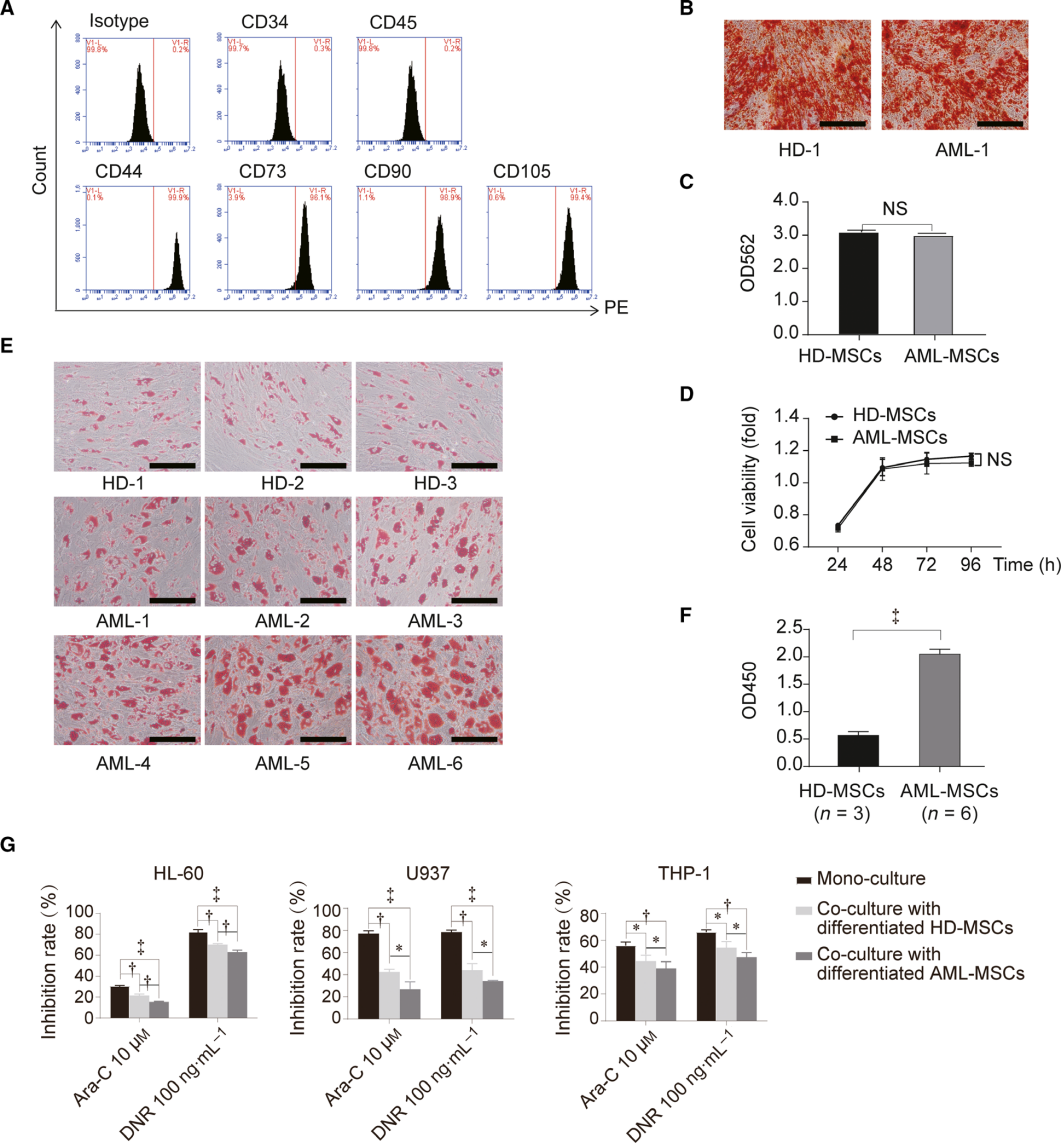
Adipogenesis of MSCs and chemoresistance of AML cells. (A) Flow cytometry of MSC surface markers. Positive markers CD44, CD73, CD90 and CD105 and negative markers are CD34 and CD45. (B) Cells were stained with Alizarin Red S at 21 days (scale bar = 500 µm). (C) Calcified nodules were dissolved in 10% cetylpyridinium chloride and absorbance measured at 562 nm. (D) CCK8 results of HD‐MSCs and AML‐MSCs. (E) AML‐MSCs and HD‐MSCs stained at 14 days (scale bar = 500 µm). (F) After ORO staining, adipocytes were dissolved in isopropanol at room temperature, and then, absorbance was measured at 450 nm. (G) The adipogenesis of HD‐MSCs and AML‐MSCs was induced for 14 days. Thereafter, adipocytes were co‐cultured with AML cells and then chemoresistance of AML cells was determined. Three independent replicates of all biological samples were assessed. The error bars represent SD. We conducted statistical comparisons using Student's *t*‐test for quantitative measures. *P < 0.05; ^†^ *P* < 0.01; ^‡^ *P* < 0.001. NS, not significant.

We harvested MSCs and induced their differentiation *in vitro*. The isolated MSCs were verified by flow cytometry as being positive for CD44, CD73, CD90 and CD105, but negative for the haematopoietic markers CD34 and CD45 (Fig. 1A). To identify the potential ability of multidirectional differentiation, MSCs were further induced into adipocytes and osteocytes, which were identified by staining with ORO and Alizarin Red S, respectively. The osteogenesis (Fig. 1B,C) and proliferation during culture (Fig. 1D) did not significantly differ between HD‐MSCs and AML‐MSCs. The capacity for adipogenesis was greater for AML‐MSCs than HD‐MSCs (Fig. 1E,F). Adipogenesis of the HD‐MSCs and AML‐MSCs was induced for 14 days, and then, the cells were co‐cultured with different AML cells to evaluate chemoresistance of the AML cells. The results showed that chemoresistance of the AML (including HL‐60, U937 and THP‐1) cells was promoted more by AML‐MSCs and then HD‐MSCs (Fig. 1G). This indicated that the enhanced adipogenesis of MSCs promotes AML cell resistance. Therefore, understanding the molecular mechanisms affecting adipogenesis is particularly significant.

### *AKT1*‐mRNA expression was increased in AML‐MSCs and promoted MSC adipogenesis

**Fig. 2:**
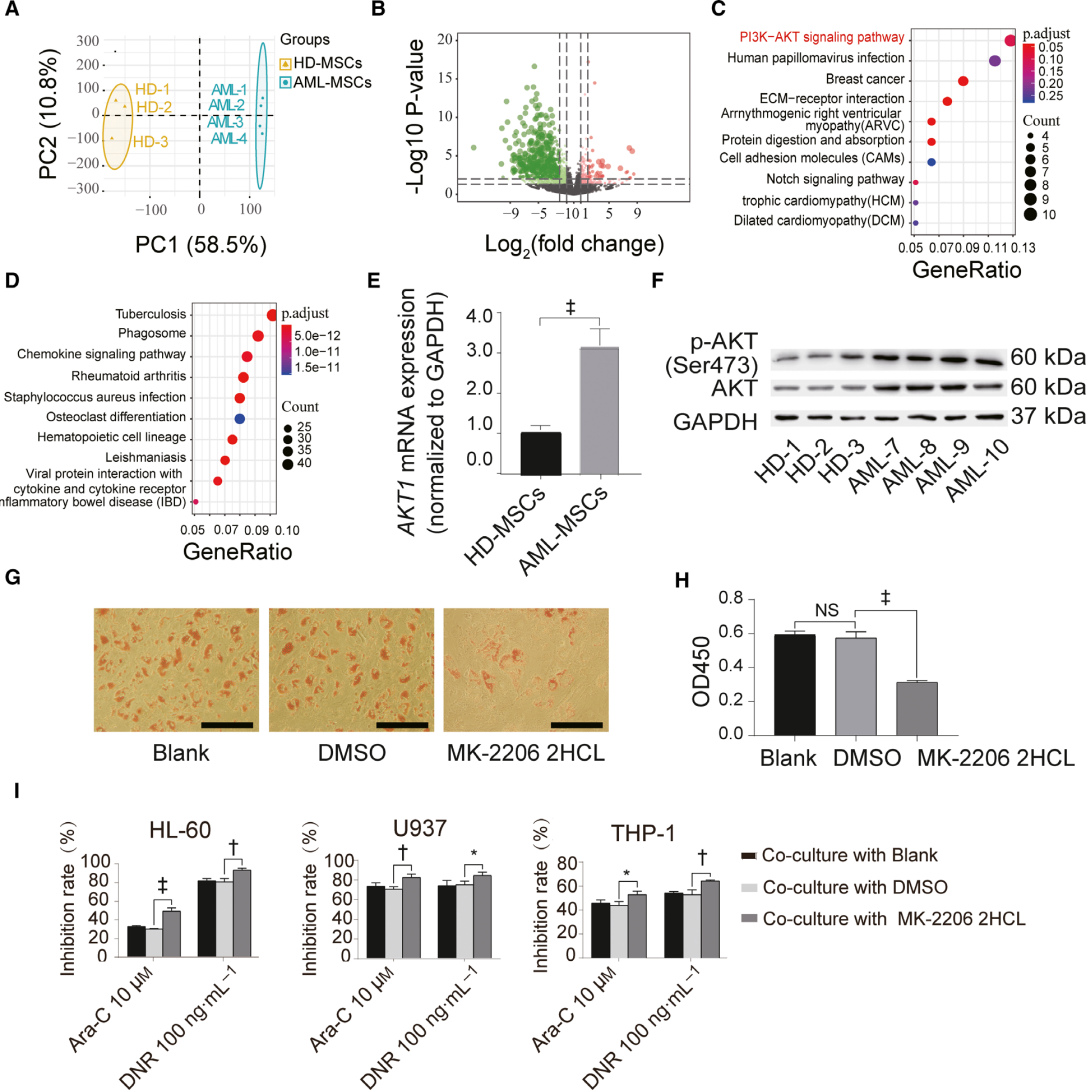
PI3K/AKT signalling pathway is expressed in AML‐MSCs and abundant AKT1 expression is associated with adipogenesis. (A) Principal component (PC) of transcription differences shows significant differences between AML‐MSCs and HD‐MSCs. The correlation variance of PC1 and PC2 are 58.5% and 10.8%, respectively. (B) Volcano map of DEGs. (C) KEGG analysis of AML‐MSCs and HD‐MSCs at level of transcription indicating significantly upregulated pathways. (D) KEGG analysis of AML‐MSCs and HD‐MSCs at level of transcription indicating significantly downregulated pathways. (E) Differences in *AKT1‐mRNA* levels between AML‐MSCs and HD‐MSCs verified by qPCR. The reference is *GAPDH* mRNA. (F) Differences in total AKT protein levels and p‐AKT (ser473) activation determined by western blotting. (G) Adipogenesis induced by AKT inhibitors (scale bar = 500 µm). (H) Isopropanol lipolysis. (I) Chemoresistance of induced AML cells to Ara‐C and DNR after co‐cultured with AML cells for 24 h. Three independent replicates of all biological samples were assessed. The error bars represent SD. We conducted statistical comparisons using Student's *t*‐test for quantitative measures. *P < 0.05; ^†^ *P* < 0.01; ^‡^ *P* < 0.001. NS, not significant.

We compared RNA sequences between MSCs from three healthy donors and four patients who were newly diagnosed with AML to identify differentially expressed genes (DEGs) between HD‐MSCs and AML‐MSCs using principal component analysis. The results revealed distinct clustering of individual HD‐MSCs and AML‐MSCs (Fig. 2A), indicating the rigour of the samples. The results of the analysis showed that 1069 genes were differentially expressed between the groups; 242 and 828 genes were, respectively, upregulated and downregulated (|fold change| ≥ 1.0; *P* < 0.05; Fig. 2B). Based on the DEGs, we analysed gene enrichment using KEGG pathways. We found that PI3K/AKT signal pathways were significantly upregulated in the AML‐MSCs compared with HD‐MSCs, which might be associated with the processes of enhancing adipogenesis (Fig. 2C). Downregulated pathways were mainly enriched in chemokine signalling, osteoclast differentiation and others pathways (Fig. 2D). The qPCR and western blotting results showed significantly upregulated AKT and p‐AKT (Ser473) expression in AML‐MSCs compared with HD‐MSCs (Fig. 2E,F). The AKT inhibitor MK‐2206 2HCL significantly reduced MSC adipogenesis (Fig. 2G,H), indicating that AKT is essential for adipogenesis of MSCs. We then incubated AML‐MSCs with MK‐2206 2HCL and induced them to differentiate into adipocytes. Co‐culture of these cells prevented chemoresistance in AML cells (Fig. 2I).

### AML‐MSCs displayed decreased global m^6^A levels and expressions of METTL3 compared with HD‐MSCs

**Fig. 3:**
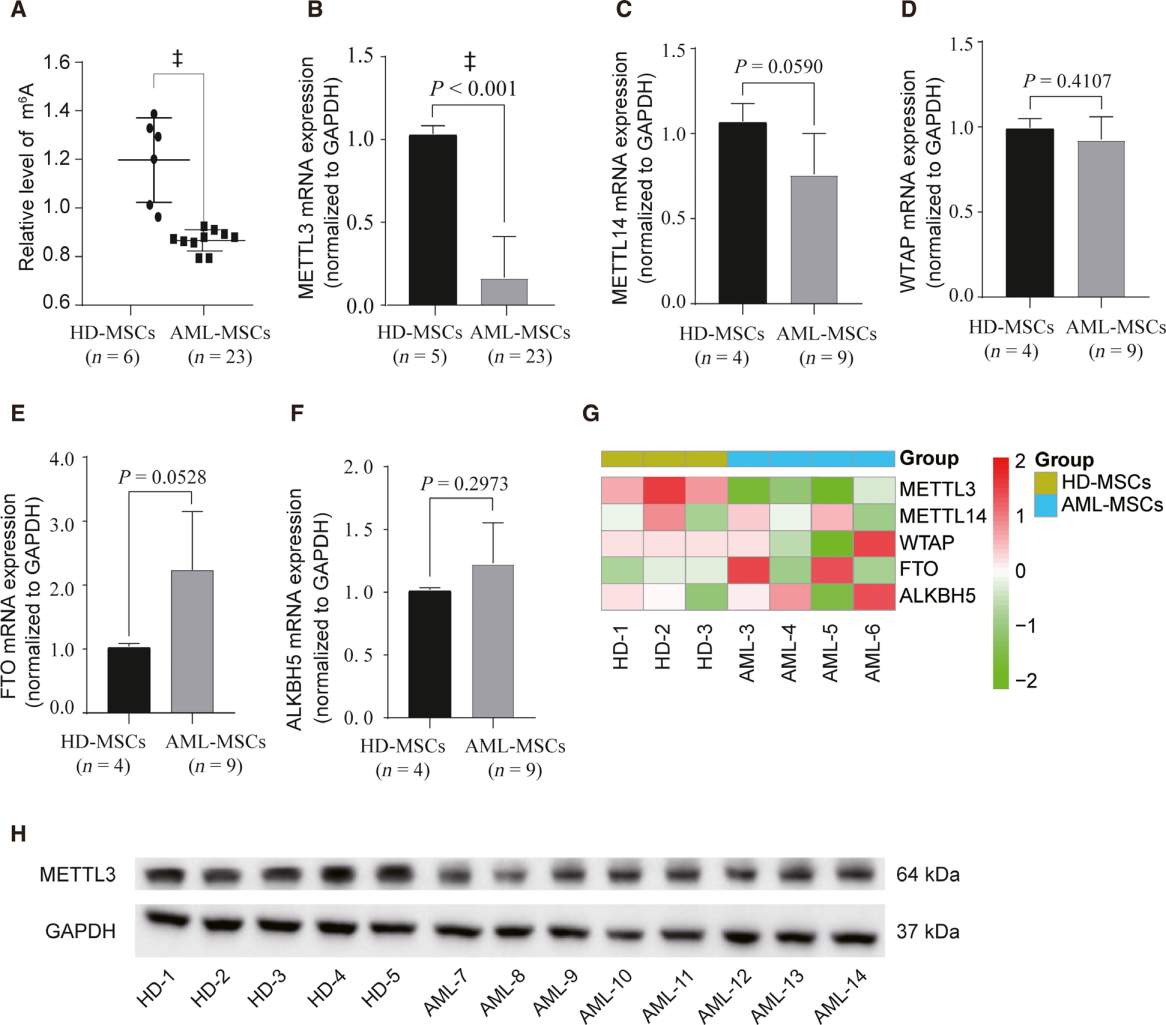
Global m^6^A levels and decreased METTL3 expression in AML‐MSCs. (A) Detections of m^6^A levels in total RNA isolated from HD‐MSCs and AML‐MSCs using EpiQuik m^6^A RNA Methylation Quantification Kits. (B–F) Relative expression of METTL3, METTL14, WTAP, FTO and ALKBH5 was determined by qPCR in HD‐MSCs and AML‐MSCs. The reference is *GAPDH* mRNA. (G) Finding of RNA sequencing shows METTL3 mRNA expression in HD‐MSCs and AML‐MSCs. (H) Relative expression of METTL3 in HD‐MSCs and AML‐MSCs was determined by western blotting. Three independent replicates of all biological samples were assessed. The error bars represent SD. We conducted statistical comparisons using Student's *t*‐test for quantitative measures. ^‡^ *P* < 0.001; NS, not significant.

The epigenetic modification RNA plays key roles in the stem cell differentiation. The m^6^A methylase METTL3 is important in the adipogenesis of BMSCs in pigs [18] and mice [19]. Therefore, we aimed to determine whether m^6^A modifications play important roles in the differentiation of human BM MSCs. Global m^6^A levels were decreased in total RNA isolated from AML‐MSCs compared with HD‐MSCs (Fig. 3A). We evaluated levels of the m^6^A‐related enzymes, METTL3, METTL14, WTAP, FTO and ALKBH5 in MSCs, and found significant differences in the expression of METTL3 (Fig. 3B–F). The founding of qPCR and western blotting revealed significantly decreased METTL3 expression in the AML‐MSCs (Fig. 3B,G). The expression of METTL3 mRNA was obvious in the heat map of RNA‐seq data. The expression of METTL3 among the five m^6^A‐related enzymes was significantly lower in AML‐MSCs than HD‐MSCs (Fig. 3H).

### Decreased METTL3 expression promoted MSC adipogenesis through an increase in AKT1

**Fig. 4:**
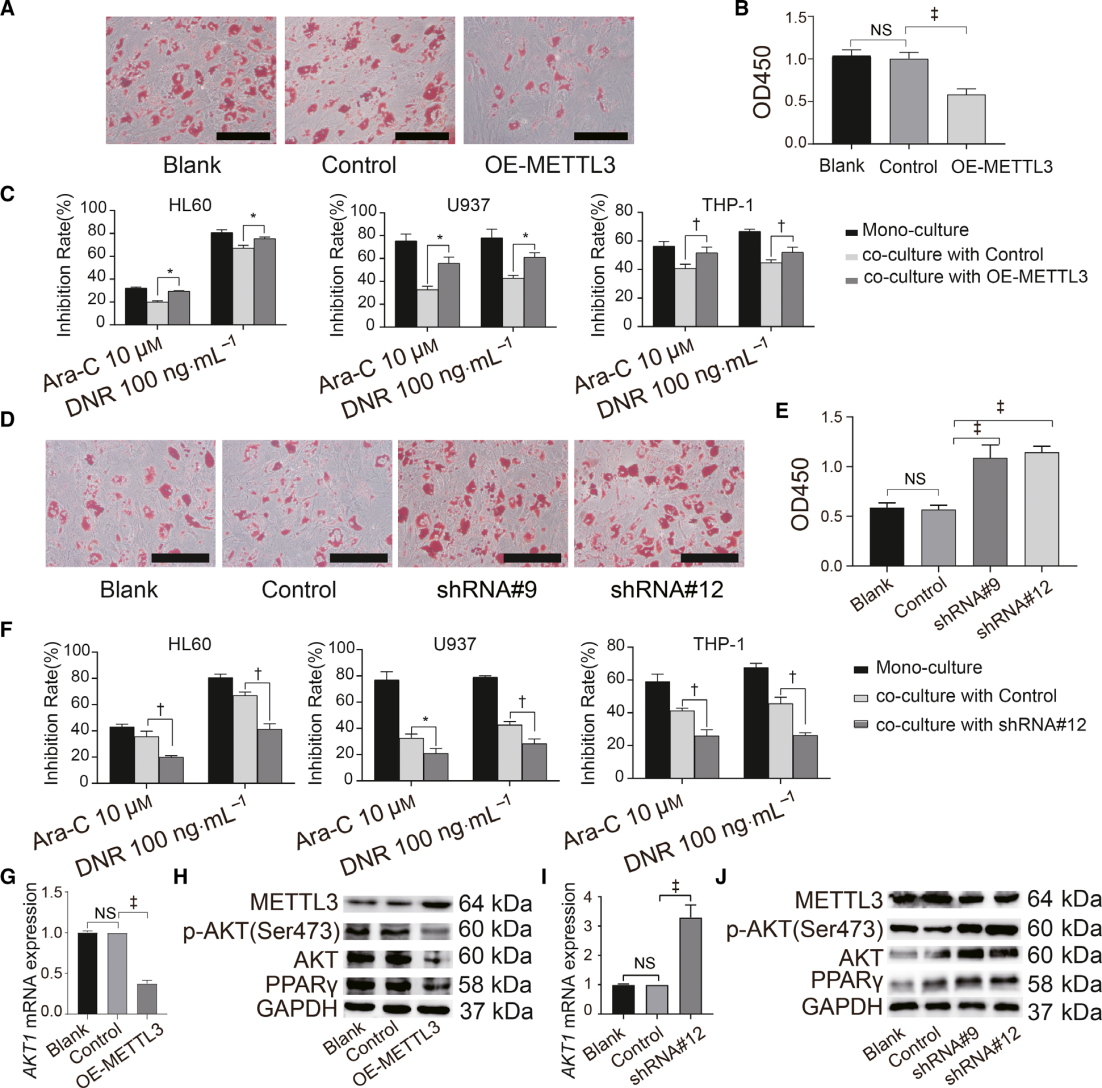
Decreased METTL3 expression promotes MSC adipogenesis by increasing AKT1. (A) Induced adipocytes stained with ORO assessed by microscopy (scale bar = 500 µm). (B) Absorbance at OD450 determined in isopropanol at room temperature. (C) Overexpressed METTL3 in AML‐MSCs induces adipogenesis. Induced cells were co‐cultured with AML cells for 24 h, and then, chemoresistance of AML cells to Ara‐C and DNR was determined. (D) Transfected HD‐MSCs with METTL3 knockdown stained with ORO after adipogenesis induction and examined under microscopy (scale bar = 500 µm). (E) Absorbance at OD450 was determined in isopropanol at room temperature. (F) METTL3 expression was knocked down in AML‐MSCs, and then, adipogenesis was induced. Adipocytes were co‐cultured with AML cells for 24 h and chemoresistance of the AML cells to Ara‐C, and DNR was determined. (G) qPCR was used to verify mRNA levels of *AKT1* after METTL3 overexpression. (H) Western blotting was used to verify protein levels of METTL3, p‐AKT, AKT and PPAR‐γ after METTL3 overexpression. (I) qPCR was used to verify mRNA levels of *AKT1* after METTL3 knockdown. (J) Western blotting was used to verify protein levels of METTL3, p‐AKT, AKT and PPAR‐γ after METTL3 knockdown. Blank, blank control; control, empty plasmid; OE, overexpression; shRNA#9 and shRNA#12, independent shRNAs targeting human METTL3. Three independent replicates of all biological samples were assessed. The error bars represent SD. We conducted statistical comparisons using Student's *t*‐test for quantitative measures. *P < 0.05; ^†^ *P* < 0.01; ^‡^ *P* < 0.001. NS, not significant.

We evaluated the adipogenesis and chemosensitivity of co‐cultured AML cells using gene editing to modulate METTL3 expression in AML‐MSCs to determine the effects of METTL3 on MSC adipogenesis. The overexpression of METTL3 significantly inhibited AML‐MSC adipogenesis (Fig. 4A,B) and increased the sensitivity of co‐cultured AML cells to chemotherapy (Fig. 4C). In contrast, METTL3 knockdown promoted AML‐MSC adipogenesis (Fig. 4D,E), and co‐culturing AML with differentiated adipocytes knocked down METTL3‐induced resistance to chemotherapy (Fig. 4F).

We further investigated the molecular mechanism through which METTL3 is linked with AML‐MSC adipogenesis. The overexpression and knockdown of METTL3, respectively, decreased and increased AKT1 expression at the mRNA level. The overexpression of METTL3 upregulated the protein expression of p‐AKT and AKT and downregulated that of PPAR‐γ (Fig. 4G,H), whereas METTL3 knockdown of exerted the opposite effects (Fig. 4I,J).

Overall, these results showed that METTL3 expression negatively regulates MSC adipogenesis and AML chemoresistance.

### METTL3 mediated AKT expression by m^6^A modification to inhibit MSC adipogenesis

**Fig. 5:**
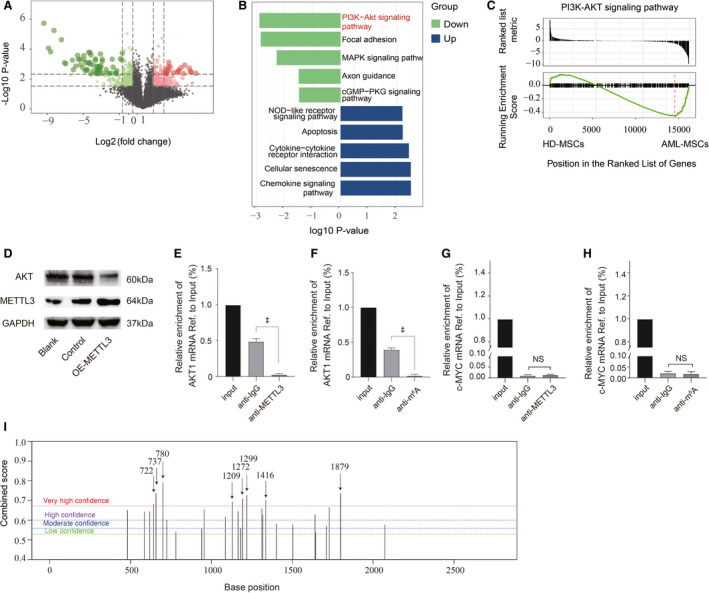
METTL3 mediates m^6^A to significantly downregulate AKT expression in AML‐MSCs. (A) Volcano map shows genes with different m^6^A levels in AML‐MSCs and HD‐MSCs. (B) Results of KEGG show pathways with different m^6^A levels in AML‐MSCs and HD‐MSCs. (C) Results of GSEA indicate changes in m^6^A levels in mRNAs of PI3K/AKT pathway genes in AML‐MSCs and HD‐MSCs. (D) Protein expression of AKT decreased after METTL3 overexpression in MCSs. (E) RT‐qPCR for *AKT1* mRNA was performed on RNA‐IP with the anti‐IgG and anti‐METTL3 antibodies. (F) m^6^A modification of *AKT1* mRNA was detected by MeRIP‐qPCR analysis using anti‐IgG and anti‐m^6^A antibodies. Relative m^6^A enrichment of *AKT1* mRNA for each IP group was normalised to input. (G) RT‐qPCR for *c‐MYC* mRNA was performed on RNA‐IP with the anti‐IgG and anti‐METTL3 antibodies. (H) m^6^A modification of *c‐MYC* mRNA was detected by MeRIP‐qPCR analysis using anti‐IgG and anti‐m^6^A antibodies. (I) Potential m^6^A sites in full‐length AKT gene predicted using SRAMP. Arrows and numbers: base positions corresponding to the *AKT1‐mRNA*. Three independent replicates of all biological samples were assessed. The error bars represent SD. We conducted statistical comparisons using Student's *t*‐test for quantitative measures. ^‡^ *P* < 0.001. NS, not significant.

We investigated possible targets of METTL3 during MSC adipogenesis by profiling m^6^A‐methylated RNAs in HD‐MSCs and AML‐MSCs using a microarray of probes for 44,122 mRNAs and 12,496 lncRNAs. We found that 127 mRNAs were differentially weakly methylated between the HD‐MSCs and AML‐MSCs (Fig. 5A). In addition, the results of KEGG pathways (Fig. 5B) and GSEA (Fig. 5C) showed that the m^6^A levels in mRNAs of genes related to the PI3K/AKT signalling pathways were significantly reduced in the AML‐MSCs compared with HD‐MSCs.

To further explore the mechanism of METTL3 mediating the regulation of AKT expression by METTL3 m^6^A to inhibit MSC adipogenesis, we overexpressed METTL3 in MSCs. Subsequent western blot findings showed that the protein expression of AKT was remarkably reduced (Fig. 5D). Further RIP assay revealed that METTL3 could bind to *AKT1‐mRNA* in MSCs (Fig. 5E). Meanwhile, RIP data indicated that *AKT1‐mRNA* in MSCs was modified with m^6^A (Fig. 5F). In the MSC cells, neither METTL3 bound to *c‐MYC* nor was *c‐MYC* present in m^6^A IPs (Fig. 5G,H). We selected *c‐MYC* as a negative control mRNA, which could rule out false positives of METTL3/m^6^A IPs. The above results indicated that METTL3 could affect the protein expression of AKT in MSCs by mediating the m^6^A modification of *AKT1‐mRNA*.

We also predicted m^6^A sites in *AKT1‐mRNA* using SRAMP. The results revealed 27 m^6^A sites (Fig. 5I) of which eight, seven and three were high‐, moderate‐ and low‐confidence sites (Table 2). Most m^6^A sites were concentrated in the CDS of *AKT1‐mRNA*. Further investigation is needed to determine which of the predicted sites are functional.

**Table 2:** Predictions of the AKT m^6^A sites.

No.	Position	Sequence context	Decision
1	561	AAGGAGCGGCCGCAGGAUGUGGACCAACGUGAGGCUCCCCUCAAC	m6A site (high confidence)
2	665	CAUCAUCCGCUGCCUGCAGUGGACCACUGUCAUCGAACGCACCUU	m6A site (high confidence)
3	698	CGAACGCACCUUCCAUGUGGAGACUCCUGAGGAGCGGGAGGAGUG	m6A site (high confidence)
4	722	UCCUGAGGAGCGGGAGGAGUGGACAACCGCCAUCCAGACUGUGGC	m6A site (very high confidence)
5	737	GGAGUGGACAACCGCCAUCCAGACUGUGGCUGACGGCCUCAAGAA	m6A site (very high confidence)
6	780	AAGCAGGAGGAGGAGGAGAUGGACUUCCGGUCGGGCUCACCCAGU	m6A site (very high confidence)
7	804	UUCCGGUCGGGCUCACCCAGUGACAACUCAGGGGCUGAAGAGAUG	m6A site (moderate confidence)
8	860	GGCCAAGCCCAAGCACCGCGUGACCAUGAACGAGUUUGAGUACCU	m6A site (low confidence)
9	1020	GUGGCCCACACACUCACCGAGAACCGCGUCCUGCAGAACUCCAGG	m6A site (low confidence)
10	1035	ACCGAGAACCGCGUCCUGCAGAACUCCAGGCACCCCUUCCUCACA	m6A site (high confidence)
11	1167	CGGGAGCGUGUGUUCUCCGAGGACCGGGCCCGCUUCUAUGGCGCU	m6A site (high confidence)
12	1209	GCUGAGAUUGUGUCAGCCCUGGACUACCUGCACUCGGAGAAGAAC	m6A site (very high confidence)
13	1245	GAGAAGAACGUGGUGUACCGGGACCUCAAGCUGGAGAACCUCAUG	m6A site (high confidence)
14	1260	UACCGGGACCUCAAGCUGGAGAACCUCAUGCUGGACAAGGACGGG	m6A site (moderate confidence)
15	1272	AAGCUGGAGAACCUCAUGCUGGACAAGGACGGGCACAUUAAGAUC	m6A site (very high confidence)
16	1299	GACGGGCACAUUAAGAUCACAGACUUCGGGCUGUGCAAGGAGGGG	m6A site (very high confidence)
17	1392	CUGGCCCCCGAGGUGCUGGAGGACAAUGACUACGGCCGUGCAGUG	m6A site (high confidence)
18	1398	CCCGAGGUGCUGGAGGACAAUGACUACGGCCGUGCAGUGGACUGG	m6A site (high confidence)
19	1416	AAUGACUACGGCCGUGCAGUGGACUGGUGGGGGCUGGGCGUGGUC	m6A site (very high confidence)
20	1482	CGCCUGCCCUUCUACAACCAGGACCAUGAGAAGCUUUUUGAGCUC	m6A site (moderate confidence)
21	1584	CUUUCAGGGCUGCUCAAGAAGGACCCCAAGCAGAGGCUUGGCGGG	m6A site (moderate confidence)
22	1721	CAAGCCCCAGGUCACGUCGGAGACUGACACCAGGUAUUUUGAUGA	m6A site (high confidence)
23	1725	CCCCAGGUCACGUCGGAGACUGACACCAGGUAUUUUGAUGAGGAG	m6A site (low confidence)
24	1791	AUCACACCACCUGACCAAGAUGACAGCAUGGAGUGUGUGGACAGC	m6A site (moderate confidence)
25	1809	GAUGACAGCAUGGAGUGUGUGGACAGCGAGCGCAGGCCCCACUUC	m6A site (high confidence)
26	1879	GCACGGCCUGAGGCGGCGGUGGACUGCGCUGGACGAUAGCUUGGA	m6A site (very high confidence)
27	2153	UUCACGUAGGGAAAUGUUAAGGACUUCUGCAGCUAUGCGCAAUGU	m6A site (moderate confidence)

Note:

The underlined text represents the m6A sites.

## Discussion

**Fig. 6:**
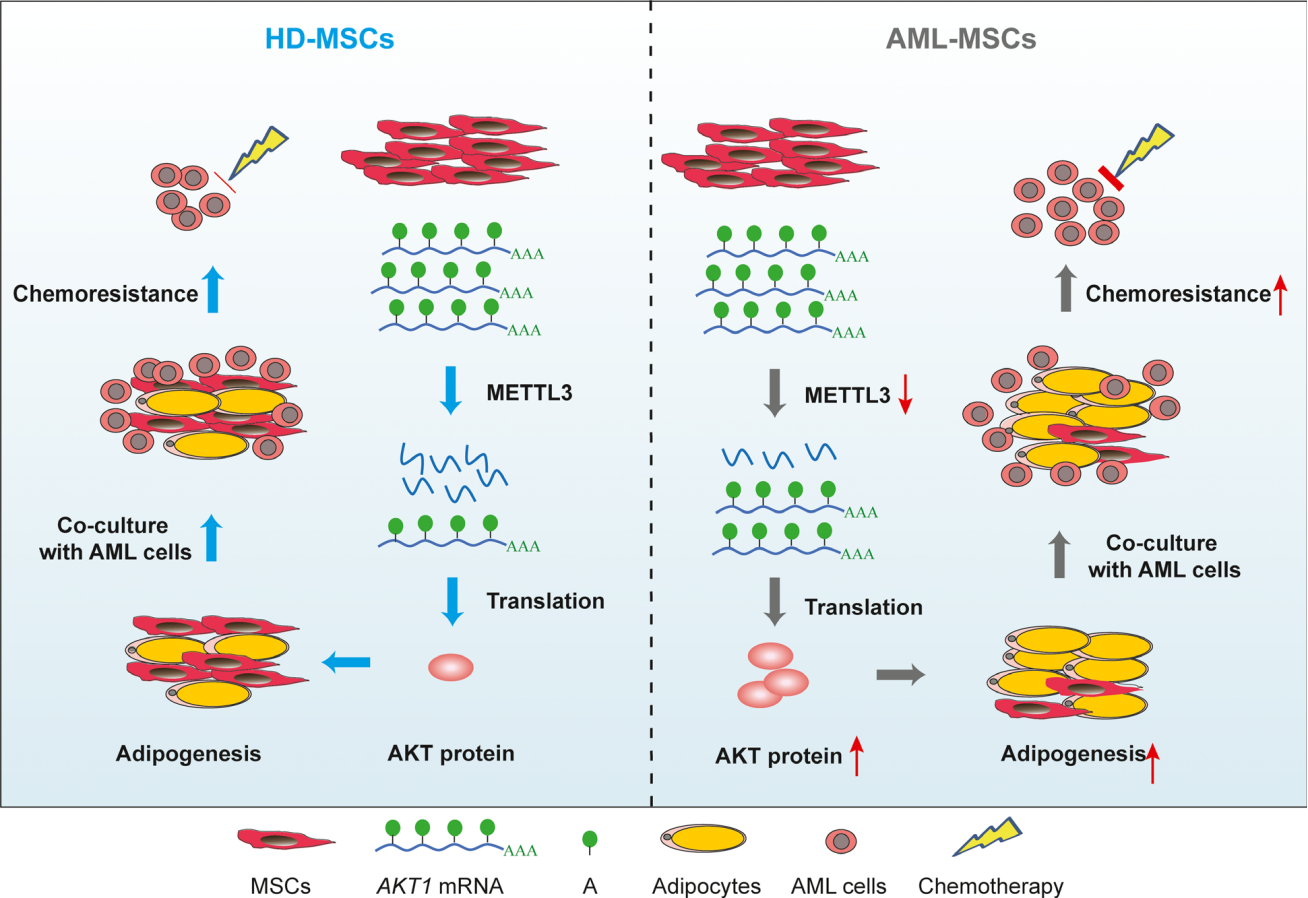
Proposed model of METTL3 regulation and role in MSC adipogenesis. Lower METTL3 expression increases AKT protein expression that promotes MSC adipogenesis and AML chemoresistance in AML‐MSCs compared with HD‐MSCs.

The BMMs of AML are remodelled to ensure that AML cells survive and resist the effects of chemotherapy. Adipocytes in BM are mainly differentiated from MSCs. The enhanced adipogenesis of AML‐MSCs can prevent chemotherapy from killing AML cells. Modification of RNA by m^6^A is the most abundant RNA modification in eukaryotic mRNAs, but the role of m^6^A‐mRNA in tumorigenesis and tumour development has not been investigated from the perspective of the tumour microenvironment of MSCs. To utilise the restructured BMM that controls the differentiation of MSCs into specific lineages for clinical AML treatment, deeper understanding of the molecular mechanism involved in specific lineage differentiation is essential. The present study identified an important mechanism that promotes the differentiation of pluripotent MSCs into adipocytes. We found that METTL3 mediates the m^6^A modification of *AKT1‐mRNA*, leading to increased *AKT1‐mRNA* and protein expression, which renders MSCs more likely to differentiate into adipocytes, thus changing the BMM and causing changes in AML chemoresistance (Fig. 6).

The dynamic and reversible m^6^A modification of RNA is the most abundant internal RNA modification in eukaryotes [32], and it plays a key role in regulating the proliferation, metastasis, pluripotency and immunity of tumour stem cells. The m^6^A methylase, METTL3, has two‐way effects in different cancers. For example, abundant METTL3 expression in AML [15, 33, 34], liver cancer [26] and glioblastoma [35] promotes the occurrence and development of tumours. In contrast, METTL3 can serve as a tumour suppressor to inhibit the growth and invasion of ovarian [36] and prostate [37] cancer. Abundant METTL3 expression inhibits MSC adipogenesis in pigs and mice. However, the effects of METTL3 on human MSCs have not been investigated in detail. The present findings showed more adipogenesis and lower METTL3 expression in AML‐MSCs than HD‐MSCs. The negative impact of METTL3 on MSC adipogenesis implied that METTL3 m^6^A‐dependently regulates the differentiation of MSCs.

The PI3K/AKT pathway plays important roles in mediating the proliferation, apoptosis and differentiation of cells [38]. Overactivation of the PI3K/AKT pathway results in aberrant cell cycle progression, altered cell adhesion and motility, inhibition of apoptosis and the induction of angiogenesis [38, 39]. Neprilysin accelerates adipogenesis in the MSC line C3H10T1/2 by enhancing PI3K/AKT activation [40]. Collectively, our results indicated that AKT plays a regulatory role in MSC adipogenesis and that m^6^A‐dependently interacts with METTL3. The preliminary results of RNA‐seq and the m^6^A microarray showed reduced m^6^A activity in the PI3K/AKT signalling pathway. The m^6^A modification of mRNAs associated with PI3K/AKT signalling pathways was significantly upregulated, which might have promoted the adipogenic differentiation of MSCs. However, the precise molecular mechanisms require further exploration. Decreases in METTL3 regulate AKT activities and promote the proliferation and tumorigenicity of endometrial cancer, and m^6^A methylation regulates AKT pathways [41]. However, METTL3 regulation of PI3K/AKT signalling pathways in the adipogenic differentiation of MSCs has not been assessed.

The effects of m^6^A modification on mRNA transcription are mediated by specific m^6^A‐binding proteins called m^6^A readers [42]. The YTH domain family of proteins bind as m^6^A in mammals [43]. For instance, YTHDF2 recognises and destabilises mRNA containing m^6^A [44]. Because m^6^A modifications and the YTH domain family are widespread in eukaryotes and play regulatory roles in various biological processes, we propose that m^6^A‐binding proteins play specific roles in m^6^A‐mediated adipogenesis. Amount of AKT protein expression negatively correlated with levels of m^6^A‐modified mRNA. We plan to further investigate whether YTHDF2 promotes AKT degradation and inhibits MSC differentiation. Due to a limited sample size, further study of a larger sample is required to verify the present finding.

We found that METTL3 is associated with the adipogenesis of human MSCs. Decreased METTL3 expression in AML‐MSCs significantly reduced the amount of m^6^A modification of mRNA associated with PI3K/AKT signalling pathways. Activation of the PI3K/AKT signalling pathways might promote MSC adipogenesis, which could potentially mediate AML chemoresistance. The present findings provide a theoretical foundation that should help to determine new targets of AML treatment from the perspective of BMMs and provide important insights that will lead to novel clinical strategies for treating AML.

## Conflicts of interest

The authors declare no conflicts of interest.

## Author contributions

H‐FH, Z‐PP and BW conceived and designed the project; ZP, DH, RY and X‐TW acquired the data; ZP and W‐HX analysed and interpreted the data; and Z‐PP and R‐LY wrote the paper.

## Data Availability

Additional data to support our conclusions are available from the corresponding author upon reasonable request.
